# Baroreflex Loading Reveals Exaggerated Ventilatory Inhibition in Patients With Parkinson's Disease

**DOI:** 10.1111/apha.70323

**Published:** 2026-10-04

**Authors:** Jhenny V. Neri, Jeann L. Sabino‐Carvalho, Bruno M. Silva, Pedro R. P. Brandão, Fabrício da Silva, Paul J. Fadel, Lauro C. Vianna

**Affiliations:** ^1^ NeuroV̇ASQ̇ – Integrative Physiology Laboratory, Faculty of Physical Education University of Brasília Brasília Brazil; ^2^ Graduate Program in Medical Sciences, Faculty of Medicine University of Brasília Brasília Brazil; ^3^ National Institute of Science and Technology‐INCT, Physical (In)activity and Health CNPq Brasília Brazil; ^4^ Department of Physiology Federal University of São Paulo São Paulo Brazil; ^5^ Department of Kinesiology University of Texas at Arlington Arlington Texas USA

**Keywords:** autonomic nervous system, baroreflex, cardiorespiratory coupling, modified Oxford technique, Parkinson's disease, ventilatory control

Did you know?

The influence of arterial baroreceptors extends beyond the short‐term regulation of arterial blood pressure (BP) to the control of breathing, an interaction recognized since the early work of Heymans and Bouckaert demonstrating that sensory mechanisms arising from the carotid sinus and aortic regions contribute to reflex respiratory regulation [1]. This work, recognized by the 1938 Nobel Prize in Physiology or Medicine, provided the foundation for subsequent studies showing that increases in BP can inhibit ventilation whereas decreases in pressure stimulate it, further supporting the concept that arterial baroreceptor input contributes to respiratory control.

The respiratory effects of arterial baroreceptor activation have subsequently been demonstrated across several species, including rodents, cats, dogs, piglets, and humans, although their magnitude and expression vary with species and experimental preparation. Early studies in humans suggested that the ventilatory inhibition accompanying pressor stimulation was mediated, at least in part, by arterial baroreceptor activation, with Heistad and colleagues showing that both phenylephrine and the nonadrenergic pressor agent angiotensin reduced minute ventilation and that the response to phenylephrine persisted during 100% oxygen, indicating that peripheral chemoreceptor mechanisms alone could not account for the response [2]. This concept was later examined more systematically by Stewart and colleagues (2011) [3] using the modified‐Oxford technique to both unload and load the arterial baroreflex, while manipulating chemoreceptor activity with room air, hypoxia, and hypercapnic conditions. Despite marked changes in baseline ventilation during peripheral and central chemoreflex activation, ventilatory baroreflex sensitivity remained unchanged, although the operating point of the pressure–ventilation relationship shifted. These findings support a baroreceptor‐mediated influence on ventilation that cannot be explained by changes in chemoreflex drive.

The neural organization underlying this response remains incompletely understood, but experimental evidence indicates that barosensory information can influence respiratory motor output through distributed medullary circuitry. In cats, arterial baroreceptor stimulation altered the activity of medullary raphe neurones while simultaneously prolonging expiration and reducing integrated phrenic activity, and functional interactions were identified between baroresponsive raphe neurones and inspiratory and expiratory neurones within the ventrolateral medulla [4]. These observations led to the proposal that medullary raphe neuronal assemblies participate in transforming and transmitting barosensory information to the ventral respiratory group, suggesting a potential neural substrate through which changes in BP may influence respiratory timing and drive. The ventrolateral medullary regions examined in these experiments included respiratory networks associated with the Bötzinger and pre‐Bötzinger complexes, further supporting the possibility that baro‐ventilatory modulation involves interactions between barosensory and respiratory networks within the medulla rather than two independent reflex systems operating in parallel [4].

This organization is potentially relevant to Parkinson's disease (PD), in which respiratory dysfunction has been linked to abnormalities in brainstem networks involved in respiratory rhythm generation, chemosensitivity, and respiratory modulation, beyond the effects of motor impairment [5]. Evidence from human and experimental studies implicates respiratory‐related regions including the pre‐Bötzinger complex, nucleus tractus solitarii, and retrotrapezoid nucleus, while neuronal loss involving an NK1 receptor‐immunoreactive population consistent with the pre‐Bötzinger phenotype has been reported in the ventrolateral medulla of patients with PD [5]. These respiratory abnormalities coexist with evidence of impaired cardiovascular baroreflex control in PD. Using the modified‐Oxford technique, cardiovagal baroreflex gain was reduced [6], while other evidence suggests that dysfunction may involve both central and peripheral components of the baroreflex arc [7]. Together, these findings raise the possibility that baro‐ventilatory integration may also be altered in PD.

To examine this possibility, we assessed baro‐ventilatory responses in eight patients with idiopathic PD (age 50 ± 5 years; 2 women; Hoehn & Yahr Stage 2 ± 1; disease duration 6 ± 3 years) and nine age‐matched controls (CT; age 50 ± 6 years; 2 women) during the modified‐Oxford technique. The present data were retrospectively reanalyzed from two previously published studies with different primary aims [6, 8]. Respiratory movements were recorded using a pneumobelt, from which respiratory rate and respiratory amplitude were derived to estimate ventilation. At rest, patients with PD exhibited a higher respiratory rate than controls (19.4 ± 1.3 vs. 13.6 ± 1.1 breaths·min^−1^), accompanied by shorter inspiratory, expiratory, and total respiratory cycle times (Ttot: 3.2 ± 0.2 vs. 4.8 ± 0.4 s; *p* = 0.008). The modified‐Oxford was accompanied by reciprocal ventilatory responses, with ventilation increasing during the drop in BP and decreasing during the subsequent rise (Figure 1A). When these responses were quantified, nitroprusside infusion produced similar reductions in BP between groups, accompanied by similar increases in estimated ventilation. In contrast, phenylephrine infusion produced similar increases in BP but greater ventilatory inhibition in PD (Figure 1B). This difference during baroreflex loading was also reflected in the pressure–ventilation relationship, with a significantly steeper slope in PD than in controls (Figure 1C). Despite this difference during baroreflex loading, the overall slope across the modified‐Oxford technique did not differ between groups (CT: −0.55 ± 0.146 vs. PD: −0.94 ± 0.264; *p* = 0.203). Similarly, during nitroprusside‐induced baroreflex unloading, pressure–ventilation slopes were comparable between groups (CT: −0.44 ± 0.060 vs. PD: −0.83 ± 0.266; *p* = 0.197).

**FIGURE 1:**
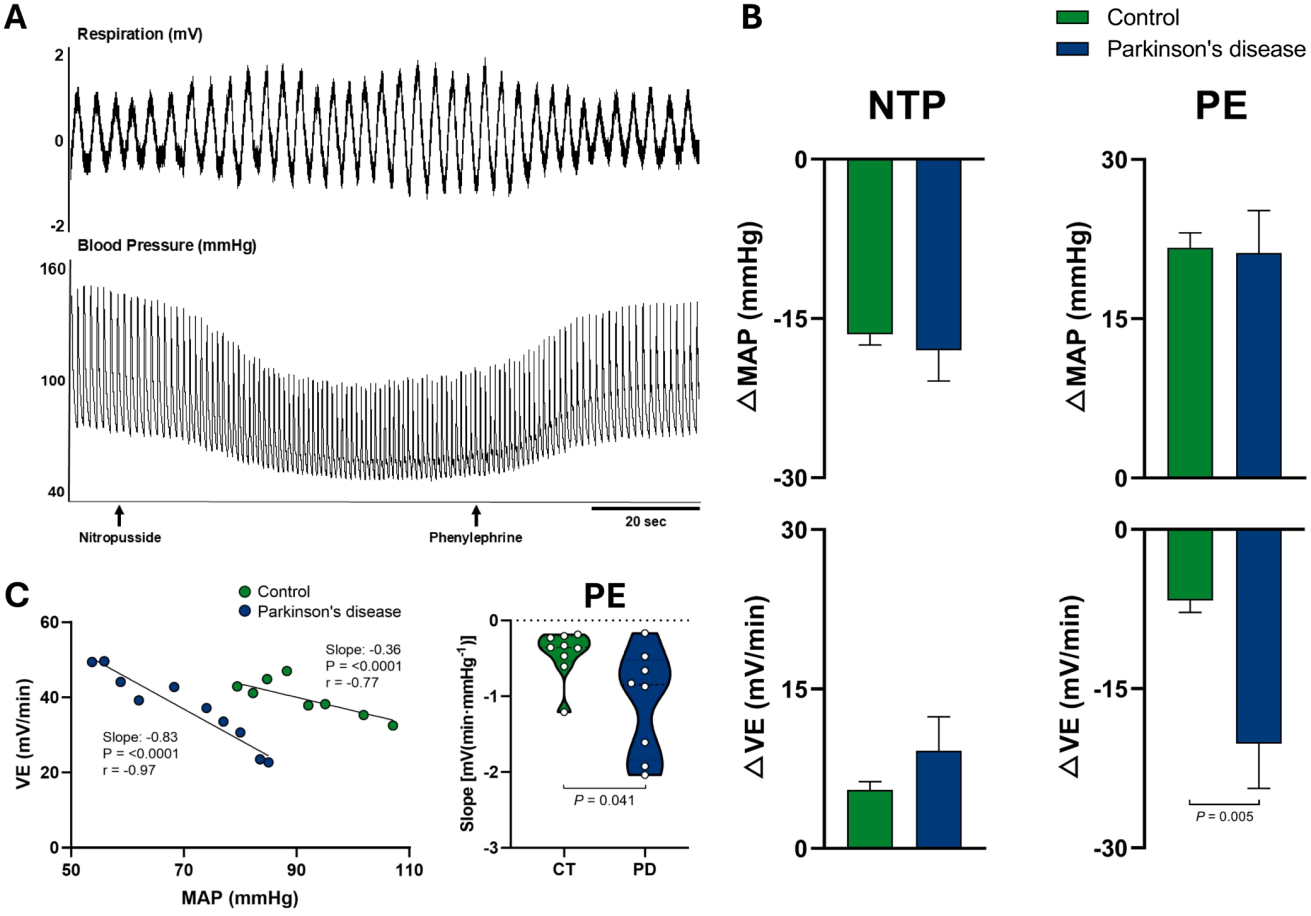
(A) Original recordings from a patient with Parkinson's disease (PD) of respiration (top) and arterial blood pressure (bottom) during the modified Oxford maneuver. (B) Changes in mean arterial pressure (ΔMAP) and estimated ventilation (ΔVE) in response to sodium nitroprusside (NTP) and phenylephrine (PE) infusion in Controls (CT; green) and PD (blue). (C) Representative pressure–ventilation slopes during PE infusion from one CT and one PD participant (left), and individual PE slopes for all participants (right). Data are presented as mean ± SEM. CT, control; PD, Parkinson's disease.

These responses across the pressure perturbation suggest that baro‐ventilatory control is altered in PD, although the magnitude of the between‐group difference was not uniform across the depressor and pressor phases. The ventilatory response during unloading was numerically greater in PD but did not provide clear statistical evidence of a group difference, whereas ventilatory inhibition during loading was markedly exaggerated. The physiological significance of this greater sensitivity remains uncertain and may reflect either an adaptive adjustment to altered cardiovascular regulation or a change in the way arterial pressure perturbations are translated into ventilatory responses. Of note, phenylephrine largely restored arterial pressure after nitroprusside‐induced hypotension rather than producing marked hypertension, yet this return toward baseline pressure was accompanied by a greater ventilatory inhibition in PD. Whether this response has compensatory relevance or contributes to altered ventilatory regulation remains to be determined.

The operating state of the respiratory network may influence baro‐ventilatory responses, as experimental studies indicate that the respiratory effects of baroreceptor activation depend on central respiratory drive. This dependence on respiratory network state may be particularly relevant to PD, in which increased airway occlusion pressure at 0.1 s (P0.1) has been reported, consistent with altered respiratory center output [9]. Thus, barosensory input may converge on a respiratory network operating from a different baseline state, although whether this contributes to the exaggerated response during baroreflex loading remains unknown. The present findings should be interpreted considering the small sample size and ongoing levodopa treatment. Ventilation was estimated from pneumobelt‐derived respiratory rate and amplitude rather than measured directly; however, this approach has been previously validated and also allows for less disruption of natural breathing patterns compared with wearing a mask or mouthpiece [10]. Nevertheless, the present findings suggest that baro‐ventilatory control may also be altered in PD, extending previous evidence of cardiac baroreflex dysfunction in this disease [6, 7]. Whether this reflects a broader disturbance of baroreflex integration in PD remains to be determined.

## Funding

J.V.N. receives scholarship support from the National Council for Scientific and Technological Development (CNPq; grant 136561/2026‐8). J.L.S.‐C. receives research support from CNPq (315309/2025‐3 and 446406/2024‐4). L.C.V. receives research support from the CNPq (307764/2022‐2), from the National Institute of Science and Technology–INCT, Physical (In) activity and Health (CNPq: 408899/2024‐7), and from the Federal District Research Support Foundation (FAPDF: 00193‐00001619/2024‐61). P.J.F. was supported by the Moritz Chair in Geriatrics, the College of Nursing and Health Innovation, the University of Texas at Arlington.

## Ethics Statement

The present study was a retrospective secondary analysis of existing respiratory and hemodynamic recordings collected during modified‐Oxford protocols previously published. Protocols conducted at the University of Brasília were approved by the Research Ethics Committee of the University of Brasília (CAAE 67005717.2.0000.0030), whereas those conducted at the University of Missouri were approved by the University of Missouri Health Sciences Institutional Review Board. All original protocols were conducted in accordance with the Declaration of Helsinki, and all participants provided written informed consent before enrollment in the original studies.

## Conflicts of Interest

The authors declare no conflicts of interest.

## Data Availability

The data that support the findings of this study are available from the corresponding author upon reasonable request.
